# Solvent-dependent electrochemical performance of UPyMA–PETEA supramolecular gel polymer electrolytes for lithium–oxygen batteries

**DOI:** 10.1039/d6ra07677g

**Published:** 2026-10-12

**Authors:** Toshiki Aizawa, Toshiki Iwato, Hayato Takada, Kentaro Ogihara, Takahiro Ishizaki

**Affiliations:** a Materials Science and Engineering, Graduate School of Engineering and Science, Shibaura Institute of Technology Toyosu, Koto-Ku Tokyo 135-8548 Japan; b College of Engineering, Shibaura Institute of Technology Toyosu, Koto-Ku Tokyo 135-8548 Japan ishizaki@shibaura-it.ac.jp

## Abstract

Supramolecular gel polymer electrolytes (SGPEs) based on UPyMA and PETEA were prepared with 1 M LiTFSI and 10 wt% TTE using sulfolane, dimethyl sulfoxide (DMSO), *N*,*N*-dimethylacetamide (DMA), or *N*,*N*-dimethylformamide (DMF) as the solvent. Structural characterization supported polymer formation and retention of the UPy hydrogen-bonding motifs. The solvent-dependent physicochemical and electrochemical characteristics of the formulations were compared by thermal analysis, viscosity measurements, electrochemical characterization, and Li–O_2_ cell testing. Among the tested SGPEs, the sulfolane-based formulation exhibited the highest apparent anodic oxidation onset, approximately 5.5 V *vs.* Li/Li^+^ under the employed linear-sweep voltammetry conditions. At 0.05 mA cm^−2^, the DMSO-based cell delivered a full-discharge capacity of 3135 mAh g^−1^, compared with 2133 mAh g^−1^ for the sulfolane-based cell. In contrast, under capacity-limited cycling at 500 mAh g^−1^, the sulfolane-based cell showed the longest cycling among the tested formulations, with the reported trace maintaining discharge capacities above 400 mAh g^−1^ through 30 cycles. Post-cycling FE-SEM and XRD revealed cathode deposits and changes in detectable crystalline products between discharge and charge. These results demonstrate divergent solvent-dependent performance within a common SGPE framework: DMSO favored full-discharge capacity, whereas sulfolane favored extended capacity-limited cycling under the tested conditions. The findings highlight that maximizing discharge capacity and sustaining repeated cycling represent distinct considerations in solvent selection for Li–O_2_ SGPEs.

## Abstract 2

Solvent selection produces divergent performance profiles within a common UPyMA–PETEA SGPE framework: DMSO favors full-discharge capacity, whereas sulfolane favors sustained capacity-limited cycling.

## Graphical abstract

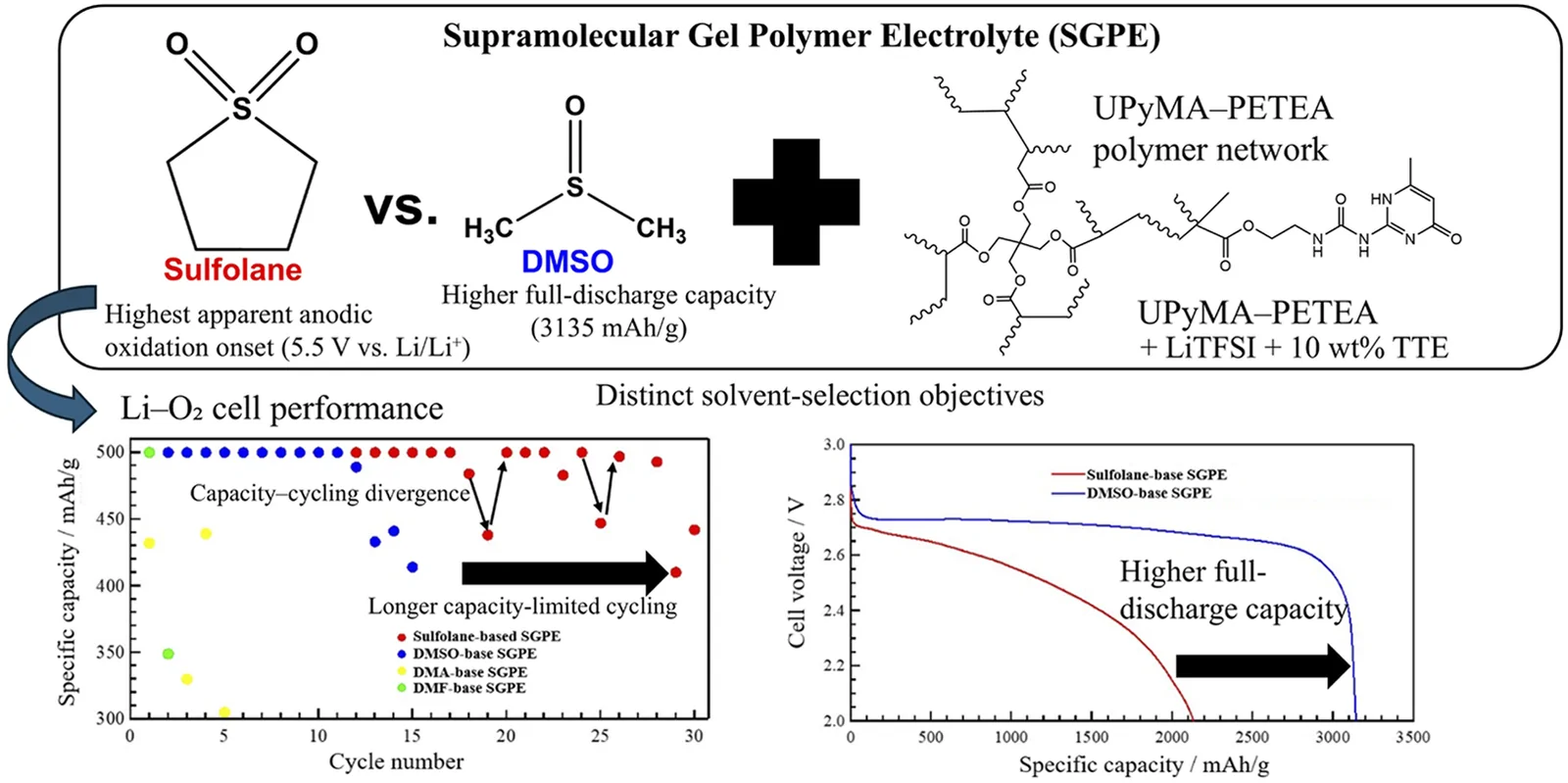

## 1 Introduction

With the global transition towards a decarbonized society, the development of next-generation energy storage devices capable of efficiently storing and utilizing renewable energy sources, such as solar and wind power, has become imperative. For the widespread adoption of electric vehicles (EVs), energy storage systems that combine energy densities surpassing those of current lithium-ion batteries (LIBs) with robust safety and durability are highly desirable. Lithium-air batteries (LABs) have garnered significant attention as promising post-LIB candidates, boasting one of the highest theoretical energy densities (∼3500 Wh kg^−1^) among rechargeable batteries, which could enable long-range driving for EVs. However, their practical implementation is hindered by numerous technical challenges, necessitating urgent research and development ranging from fundamental studies to practical applications.^1–3^ When employing aprotic organic solvents as electrolytes, the oxygen reduction reaction (ORR) during discharge and the oxygen evolution reaction (OER) during charge proceed according to the following eqn (1):

1 2Li^+^ + 2e^−^ + O_2_ ⇆ Li_2_O_2_ (*E*^0^ = 2.96 V *vs.* Li/Li^+^)

The discharge product, lithium peroxide (Li_2_O_2_), is an electrical and ionic insulator; consequently, its decomposition during the charging process requires a substantial overpotential. This high charging overpotential induces the oxidative decomposition of both the organic electrolyte and the carbon cathode, leading to the accumulation of by-products (*e.g.*, Li_2_CO_3_), which severely degrades cycle life.^4–6^ Furthermore, safety remains a critical concern when utilizing liquid aprotic electrolytes. Since many organic solvents are highly flammable, volatile, and toxic, the risks associated with solvent evaporation, leakage, and ignition are particularly elevated in LABs, which operate as open systems exposed to the atmosphere.^7^ Additionally, the use of a metallic lithium anode poses the risk of lithium dendrite growth during repeated cycling. These dendrites can penetrate the separator, leading to internal short circuits that may trigger thermal runaway or fire.^8^ These challenges have motivated the development of electrolyte systems that combine high ionic transport with reduced volatility, leakage, and susceptibility to dendrite-induced short-circuiting. To address these issues stemming from liquid electrolytes, oxide-based solid electrolytes have garnered attention. Solid electrolytes offer distinct advantages, including high chemical and electrochemical stability, non-flammability, non-volatility, and the ability to physically suppress dendrite growth due to their high mechanical strength.^9^ However, their ionic conductivity is generally lower than that of liquid electrolytes. Moreover, the extremely high interfacial resistance at the solid–solid interface with electrodes—particularly the lithium metal anode—makes achieving practical battery operation more challenging compared to liquid-based systems.^9^

Against this backdrop, research into gel polymer electrolytes (GPEs), which possess properties intermediate between liquids and solids, has intensified.^10^ By immobilizing the liquid electrolyte within a polymer matrix, GPEs can retain relatively high ionic conductivity while providing improved electrode/electrolyte contact compared with rigid solid electrolytes. Simultaneously, they offer enhanced safety through non-flammability or flame retardancy and the suppression of dendrite growth.^11–13^ This enhancement in safety and durability is a critical factor in ensuring the sustainability of the battery system. Nevertheless, conventional GPEs often remain inferior to liquid electrolytes in terms of ionic conductivity and interfacial resistance, underscoring the need for the development of high-performance GPEs.^10^

Recently, supramolecular gel polymer electrolytes (SGPEs) have been proposed as a novel class of functional materials. SGPEs are reported to achieve a balance among high mechanical strength, ionic conductivity, and excellent electrode/electrolyte interfacial properties by forming a supramolecular network *via* reversible physical cross-linking, such as hydrogen bonding, between polymer chains.^14–20^ Such reversible interactions have been proposed to help accommodate electrode volume changes and local mechanical stress, potentially contributing to improved cycling stability.^14–20^ However, while considerable attention has been directed toward polymer-network design, the properties of the liquid solvent retained within the gel remain equally important because the solvent governs ion solvation and transport and can strongly influence electrochemical and chemical reactions at both the lithium–metal anode and the oxygen cathode. In Li–O_2_ batteries, these requirements are particularly demanding because the electrolyte is exposed to reactive oxygen species and high charging potentials. Therefore, solvent selection within an SGPE should consider both transport-related properties and chemical/electrochemical stability rather than the polymer framework alone.

Recent studies have demonstrated that polymer-electrolyte performance can be improved by coordinating bulk ion transport with electrode-interface design. Duan *et al.* reported a cross-linked single-ion gel electrolyte in which anionic polymer segments contribute to Li^+^ coordination and interphase formation.^21^ Sun *et al.* investigated a semi-interpenetrating polymer network combining mechanical reinforcement, ionic conduction, and lithium–metal compatibility.^22^ Electrolyte/interphase design has also been advanced through control of salt-anion coordination and interphase mechanical integrity. Chen *et al.* demonstrated the use of anions with different coordination strengths to regulate Li^+^ transport and electrode compatibility,^23^ while Wang *et al.* enhanced SEI cohesion through an *in situ* formed polysiloxane component.^24^ These studies highlight that electrolyte performance reflects coupled contributions from ion transport, polymer-network properties, and electrode interfacial reactions. For solvent-containing SGPEs, however, the solvent itself represents another key formulation variable because changes in solvent chemistry can simultaneously alter transport, electrochemical stability, and reaction environments at the electrodes. Thus, comparing different solvents within a common polymer and additive framework provides a means of examining solvent-dependent performance without intentionally changing the underlying network design.

In this study, we investigate how solvent selection influences the physicochemical and electrochemical characteristics of UPyMA–PETEA SGPEs and their performance in Li–O_2_ batteries. UPyMA contains a ureidopyrimidinone (UPy) group capable of quadruple hydrogen bonding, whose reversible association has been well established in supramolecular polymers.^25^ PETEA was employed as a multifunctional cross-linker to construct the covalently cross-linked polymer matrix.^26^ The combination of PETEA cross-linking and UPy association sites provides a molecular design in which reversible non-covalent interactions are incorporated into a chemically cross-linked gel framework. Accordingly, the UPyMA–PETEA network provides a common supramolecular framework in which solvent effects can be compared while maintaining the same basic polymer design. Although UPy association provides the molecular basis for reversible non-covalent interactions, the present study does not directly evaluate macroscopic self-healing or mechanical recovery.

In addition, 10 wt% TTE was incorporated as a fluorinated component of the electrolyte formulation. Fluorinated interphase-forming strategies and molecular electrolyte design have been investigated as approaches for controlling electrode interfaces and transport properties in lithium-based batteries.^27,28^ TTE was therefore incorporated as a common formulation component across the four solvent systems. Its possible influence on liquid-phase properties and reactions at the lithium interface is considered in discussing the electrochemical results, while the specific physical and interphase-related contributions of TTE are not independently distinguished in the present study.

Using this common UPyMA–PETEA/LiTFSI/TTE framework, four aprotic solvents—sulfolane, dimethyl sulfoxide (DMSO), *N*,*N*-dimethylacetamide (DMA), and *N*,*N*-dimethylformamide (DMF)—were systematically compared. Thermal mass-retention behavior, liquid-formulation viscosity, apparent anodic oxidation behavior, Li^+^ transport characteristics, Li–O_2_ discharge and cycling performance, and post-cycling cathode products were evaluated. By changing the solvent while maintaining the common polymer, lithium salt, and TTE framework, this study examines how solvent-dependent physicochemical and electrochemical characteristics translate into distinct Li–O_2_ battery performance profiles. In particular, we compare full-discharge capacity with capacity-limited cycling behavior to determine whether the formulation favoring high discharge capacity also favors sustained cycling. This comparison provides a basis for discussing solvent selection as a multi-property optimization problem in supramolecular gel electrolytes for Li–O_2_ batteries.

## 2 Experimental section

### 2.1 Synthesis and characterization of UPyMA monomer

2-Amino-4-hydroxy-6-methylpyrimidine, 2-isocyanatoethyl methacrylate, pentaerythritol tetraacrylate (PETEA), azobis(isobutyronitrile) (AIBN), lithium bis(trifluoromethanesulfonyl)imide (LiTFSI), 1,1,2,2-tetrafluoroethyl-2,3,3-tetrafluoropropyl ether (TTE), sulfolane, dimethyl sulfoxide (DMSO), *N*,*N*-dimethylacetamide (DMA), and *N*,*N*-dimethylformamide (DMF) were purchased from Tokyo Chemical Industry (TCI) Co., Ltd or Sigma-Aldrich Co. LLC and used as received unless otherwise specified.

The synthetic route for 2-(3-(6-methyl-4-oxo-1,4-dihydropyrimidin-3-yl)ureido)ethyl methacrylate (UPyMA) is illustrated in Fig. 1. In this study, UPyMA was synthesized following previously reported procedures with slight modifications.^13,29,30^ First, 2-amino-4-hydroxy-6-methylpyrimidine (1.0 g) was dissolved in dimethyl sulfoxide (DMSO, 25 mL) at 150 °C with stirring for 10 min. Subsequently, 2-isocyanatoethyl methacrylate (1.2 mL) was added to the solution, followed by immediate quenching in an ice bath. The resulting white precipitate was then stirred at room temperature for 24 h to ensure the completion of the reaction. The precipitate was collected *via* vacuum filtration and washed thoroughly with acetone and methanol (at least three times each) to remove unreacted precursors and impurities. Finally, the obtained white solid was dried under vacuum at room temperature for 24 h to yield the UPyMA monomer. The chemical structure of the synthesized monomer was characterized using proton nuclear magnetic resonance spectroscopy (^1^H NMR, JNM-ECZ400R, JEOL) and Fourier transform infrared spectroscopy (FT-IR, IRTracer-100, Shimadzu).

**Fig. 1:**
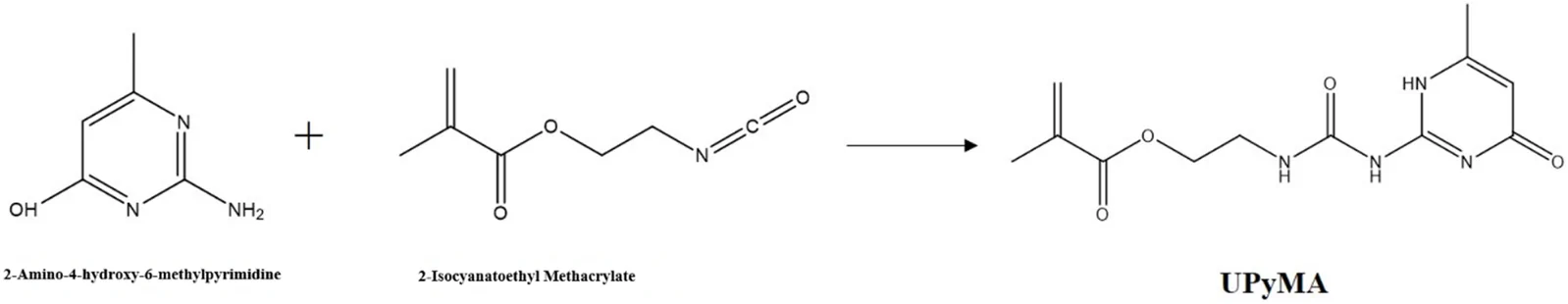
Schematic illustration of the reaction mechanism for UPyMA.

### 2.2 Preparation of SGPEs and characterization of polymer structure

The formation mechanism of the polymer matrix is illustrated in Fig. S1 of the SI.^14,19^ UPyMA was employed as a functional monomer, PETEA as a multifunctional cross-linker, and AIBN as the thermal initiator. Precursor solutions were prepared using four different aprotic organic solvents: sulfolane, dimethyl sulfoxide (DMSO), *N*,*N*-dimethylformamide (DMF), and *N*,*N*-dimethylacetamide (DMA). Lithium bis(trifluoromethanesulfonyl)imide (LiTFSI) was added to each solvent at a concentration of 1 M, and TTE was subsequently added at 10 wt%. UPyMA was then added at 3 wt%, followed by the addition of 1.5 wt% PETEA and 0.1 wt% AIBN. The resulting precursor solutions were thermally polymerized at 85 °C to form supramolecular gel polymer electrolytes (SGPEs). Literature-reported properties of the neat solvents are summarized in Table S1. The measured viscosities of the LiTFSI/TTE-containing liquid electrolyte formulations are reported separately in Table S2. Hereafter, the SGPE samples are identified according to the solvent used and are referred to as the sulfolane-, DMSO-, DMA-, and DMF-based SGPEs, respectively. For example, the SGPE prepared using the sulfolane-based electrolyte formulation containing 1 M LiTFSI and 10 wt% TTE is referred to as the “sulfolane-based SGPE”. To characterize the polymer structure, the obtained SGPEs were pulverized and ultrasonically washed in acetone for 30 min, followed by centrifugation to separate the polymer matrix from the liquid electrolyte components. The collected polymer fraction was dried under vacuum at room temperature and analyzed by Fourier-transform infrared (FT-IR; IRTracer-100, Shimadzu) spectroscopy. Details of the viscosity measurements are provided in Section S1.1 of the SI.

### 2.3 Electrochemical measurements

Linear sweep voltammetry (LSV) was conducted to compare the anodic oxidation behavior of the liquid electrolyte formulations and SGPEs. The potential was swept from 1.0 to 6.0 V *vs.* Li/Li^+^ at a scan rate of 0.1 mV s^−1^. The apparent lithium transference number (*t*_Li^+^_) was determined using the Bruce–Vincent method^31^ with Li‖Li symmetric cells (Li|SGPE|Li) assembled in an argon-filled glovebox. The symmetric cells were prepared by impregnating the glass fiber separator with the precursor solution, followed by the same *in situ* thermal polymerization process (85 °C, 30 min). The cells were allowed to rest for at least 24 h before electrochemical measurements. Initial EIS spectra were recorded over a frequency range of 0.01 Hz–1 MHz using an AC voltage amplitude of 10 mV. Subsequently, a potential step (Δ*V*) of 10 mV relative to the open-circuit voltage was applied and maintained for 7200 s, and the current response was recorded by chronoamperometry. EIS measurements were repeated after polarization using the same frequency range and AC amplitude. The initial current (*I*_0_) and the current measured after 7200 s of polarization, which was taken as the steady-state current (*I*_ss_), were obtained from the chronoamperometric response. The interfacial resistances before (*R*_0_) and after (*R*_ss_) polarization were determined by EIS fitting. The apparent Li^+^ transference number was calculated according to the Bruce–Vincent equation (eqn (2)):2
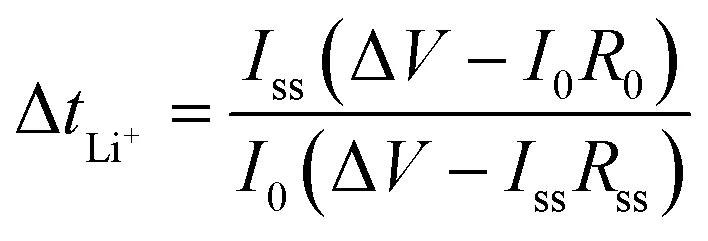
where Δ*V* is the applied polarization voltage (10 mV), *I*_0_ and *I*_ss_ represent the initial and steady-state currents, and *R*_0_ and *R*_ss_ denote the initial and steady-state interfacial resistances obtained from EIS fitting before and after polarization, respectively. Because the present measurements do not independently establish an ideal steady state and were performed in concentrated gel-electrolyte systems, the calculated values are referred to here as apparent Li^+^ transference numbers.

Because gelation was performed *in situ* within the porous separator, the effective transport thickness was not independently determined. Accordingly, absolute ionic conductivity was not calculated, and the impedance results were used for comparative evaluation of cell-level resistance. The same separator type (Whatman GF/A, nominal thickness approximately 260 µm) and precursor-solution volume were used for all symmetric cells.

Details of the preparation of the air cathode are provided in Section S1.2 of the SI.

### 2.4 Battery assembly and performance evaluation

Lithium-air battery cells were constructed using the prepared components: MWCNT-loaded carbon paper as the air cathode, a metallic lithium foil as the anode, and a glass fiber filter (GF/A, Whatman) as the separator. Precursor solutions containing each solvent (sulfolane, DMSO, DMF, or DMA) were used to form the SGPEs in the assembled cells. Cell assembly was performed in an argon-filled glovebox (O_2_ < 1 ppm, dew point <−80 °C) using split-type cells (EQ-STC-LI-AIR, MTI Japan). The assembled cells were heated in an electric furnace at 85 °C for 30 min to induce *in situ* thermal polymerization of the SGPE within the cell. Thus, gelation was performed after cell assembly, with the precursor solution present within the porous cathode and glass fiber separator. Battery performance was evaluated using a charge–discharge testing system (HJ1005SD8 or HJ1001SDC, Hokuto Denko). The tests were conducted under an oxygen flow rate of 50 mL min^−1^. Galvanostatic charge–discharge tests were performed at a current density of 0.05 mA cm^−2^ within a voltage range of 2.0–4.5 V. Specific capacities were normalized to the mass of MWCNTs loaded onto the cathode, excluding the carbon-paper substrate and PVDF binder. The current density was normalized to the geometric electrode area. The geometric area of the air cathode was 1.77 cm^2^. Cycle performance was evaluated at the same current density under capacity-limited conditions of 500 mAh g^−1^, with voltage cutoffs of 2.0 and 5.0 V for discharge and charge, respectively. Details of the characterization of the discharge products are provided in Section S1.3 of the SI.

## 3 Results and discussion

### 3.1 Structural characterization of UPyMA

The chemical structure and the ^1^H NMR spectrum of the synthesized UPyMA are presented in Fig. 2. CDCl_3_ (containing TMS as an internal standard) was used as the solvent. As shown in Fig. 2(b), the obtained spectrum is in good agreement with the chemical structure of UPyMA depicted in Fig. 2(a) and with previous reports.^14,29^ Specifically, the three singlet peaks observed in the downfield region of 10.0–13.0 ppm (a: 13.0 ppm, b: 11.9 ppm, c: 10.5 ppm) are clearly assigned to the N–H protons of the imide and urea groups associated with the UPy moiety. Furthermore, the peaks observed at 6.2 ppm (d) and 5.5 ppm (f) are assigned to the vinyl protons (CH_2_=) of the methacrylate group, while the peaks at 1.9 ppm (j) and 2.2 ppm (i) are assigned to the methyl groups (–CH_3_). The peaks at 4.3 ppm (g) and 3.6 ppm (h) correspond to the ethylene linker moiety (–OCH_2_CH_2_N–). The agreement between these characteristic resonances and the expected UPyMA structure, together with the consistency with previous reports,^14,29^ supports the successful synthesis of UPyMA containing both the polymerizable methacrylate group and the UPy moiety.

**Fig. 2:**
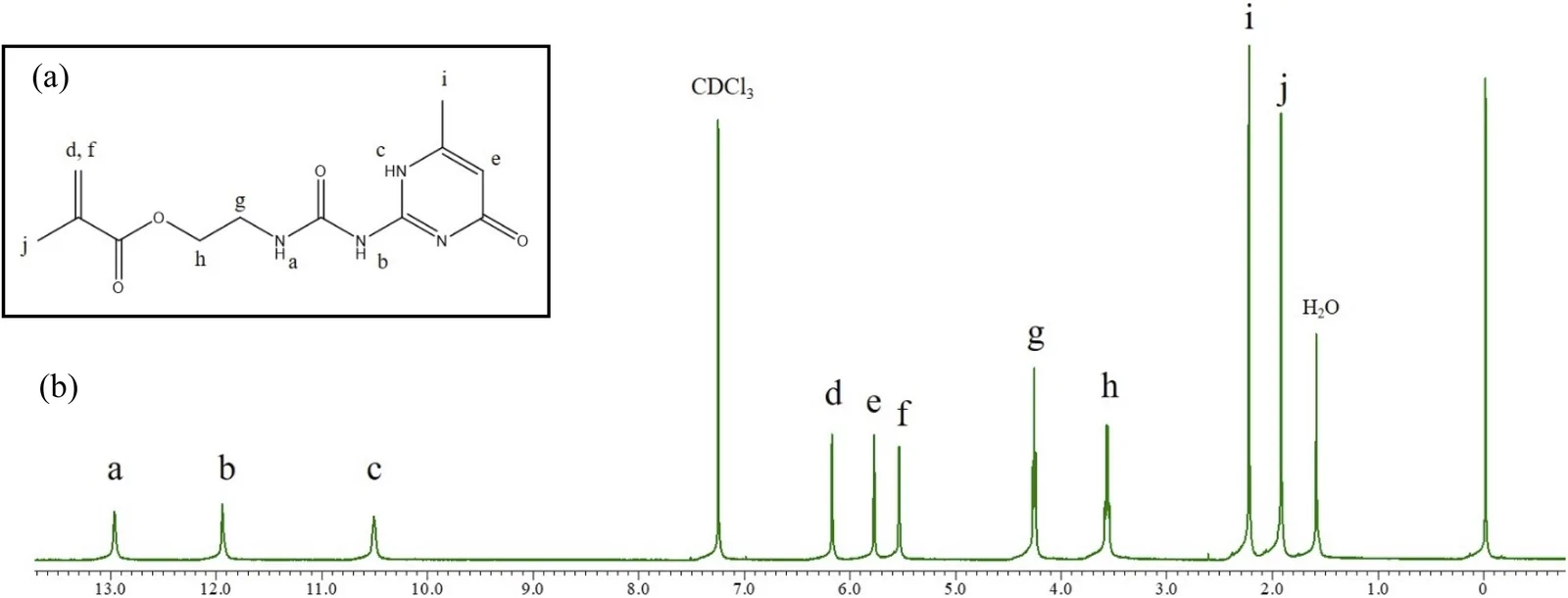
(a) Chemical structure and (b) ^1^H NMR spectrum of UPyMA.

### 3.2 Structural characterization of SGPE polymer matrix

FT-IR spectroscopy was employed to characterize the chemical structure of the polymer matrix and assess the consumption of the polymerizable C=C groups. Fig. 3 presents the FT-IR spectra of the synthesized polymer matrix, along with those of the precursors, UPyMA monomer, and PETEA. First, regarding C=C consumption during polymerization, the spectra of both UPyMA and PETEA exhibit a characteristic absorption peak at approximately 1635 cm^−1^, assigned to the C=C stretching vibration of the methacrylate and acrylate groups. In contrast, no distinct C=C absorption band at approximately 1635 cm^−1^ was detected in the spectrum of the washed polymer fraction. This change supports the consumption of polymerizable C=C groups during thermal polymerization and the formation of the cross-linked polymer matrix. Because the spectrum was recorded for the washed polymer fraction, it does not provide a quantitative measure of monomer conversion or exclude residual monomers in the as-prepared SGPE.^13,18^ Next, the structural features of the resulting polymer matrix were examined. The polymer spectrum displays strong absorption bands characteristic of the ester groups within the PETEA skeleton: the C=O stretching vibration at *ca.* 1720 cm^−1^, the C–O antisymmetric stretching at 1263 cm^−1^, and the C–O symmetric stretching at 1170 cm^−1^. These signals support the presence of PETEA-derived structural units in the polymer matrix. Furthermore, characteristic bands associated with the UPy moiety were retained after polymerization. The peaks observed at approximately 1603 cm^−1^ and 1510 cm^−1^ are assigned to the stretching vibration of the pyrimidinone ring (N=C) and the bending vibration of the ureido group (N–H), respectively. The retention of these characteristic bands indicates that UPy-derived structural motifs remain present in the polymer matrix after thermal polymerization. Combined with the ^1^H NMR results discussed in the previous section, the FT-IR results support the formation of a polymer matrix containing both PETEA-derived cross-linking units and UPy-derived functional motifs.

**Fig. 3:**
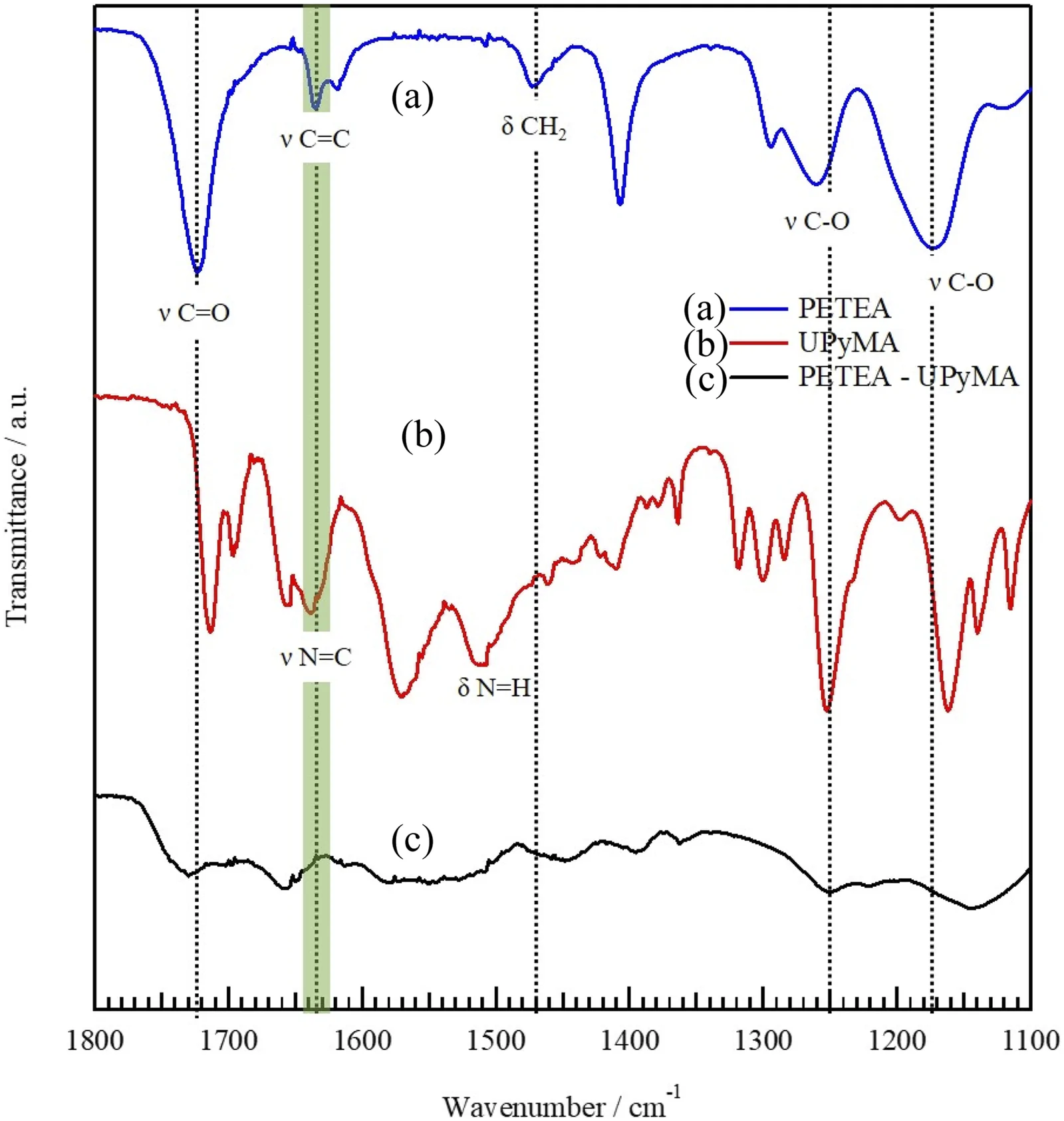
FT-IR spectra of (a) PETEA, (b) UPyMA, and (c) the washed UPyMA–PETEA polymer matrix.

The retained UPy-derived bands are consistent with preservation of the functional groups responsible for hydrogen-bond association. PETEA provides covalent cross-linking, whereas UPy groups introduce sites capable of reversible non-covalent association into the polymer framework.^14,19^ Thus, the spectroscopic results support the intended molecular design of a covalently cross-linked polymer matrix incorporating supramolecular association sites. However, FT-IR characterization alone does not establish the extent or dynamics of UPy association in the solvent-swollen SGPE, nor does it directly demonstrate macroscopic mechanical recovery or self-healing behavior.

### 3.3 Physicochemical properties of electrolytes and SGPEs

Considering the use of volatile organic electrolyte components in Li–O_2_ batteries, solvent retention and thermal behavior are important physicochemical characteristics of electrolyte formulations. Therefore, we evaluated the thermal mass-retention behavior and viscosity characteristics of liquid electrolytes and SGPEs containing different organic solvents using TG-DTA and a rotational viscometer. As a benchmark, 1 M LiTFSI in TEGDME (G4), a widely used electrolyte for LABs, was also measured.

Fig. 4(a) and (b) present the thermogravimetric analysis (TGA) curves of the various liquid electrolytes and SGPEs, respectively, over a temperature range from room temperature to 400 °C. As shown in Fig. 4(a), the liquid electrolytes exhibited significant differences in volatilization behavior depending on the solvent type. Liquid electrolyte formulations based on DMSO, DMF, and DMA exhibited pronounced mass loss in the lower-temperature region, with approximately 50 wt% or more of the initial sample mass lost by 100 °C. In contrast, the sulfolane- and G4-based formulations exhibited substantially higher mass retention, with mass losses of approximately 10 wt% or less at 100 °C. Among the tested liquid formulations, the sulfolane-based electrolyte showed the highest mass retention over this temperature region, consistent with the comparatively high boiling point of sulfolane (*ca.* 285 °C). Fig. 4(b) presents the thermogravimetric profiles of the SGPEs. Compared with the corresponding liquid formulations, the gel-containing samples showed higher mass retention in the lower-temperature region. Among the SGPEs examined, the sulfolane-based sample retained a larger fraction of its initial mass in this region than the DMSO-, DMA-, and DMF-based samples. Because the liquid and gel samples differ in composition, particularly in the presence of the polymer matrix, their normalized TGA curves cannot be used to quantify the extent to which gelation alone suppresses solvent loss. Nevertheless, the results show that the solvent-dependent differences in mass-retention behavior remain evident after formation of the SGPEs.

**Fig. 4:**
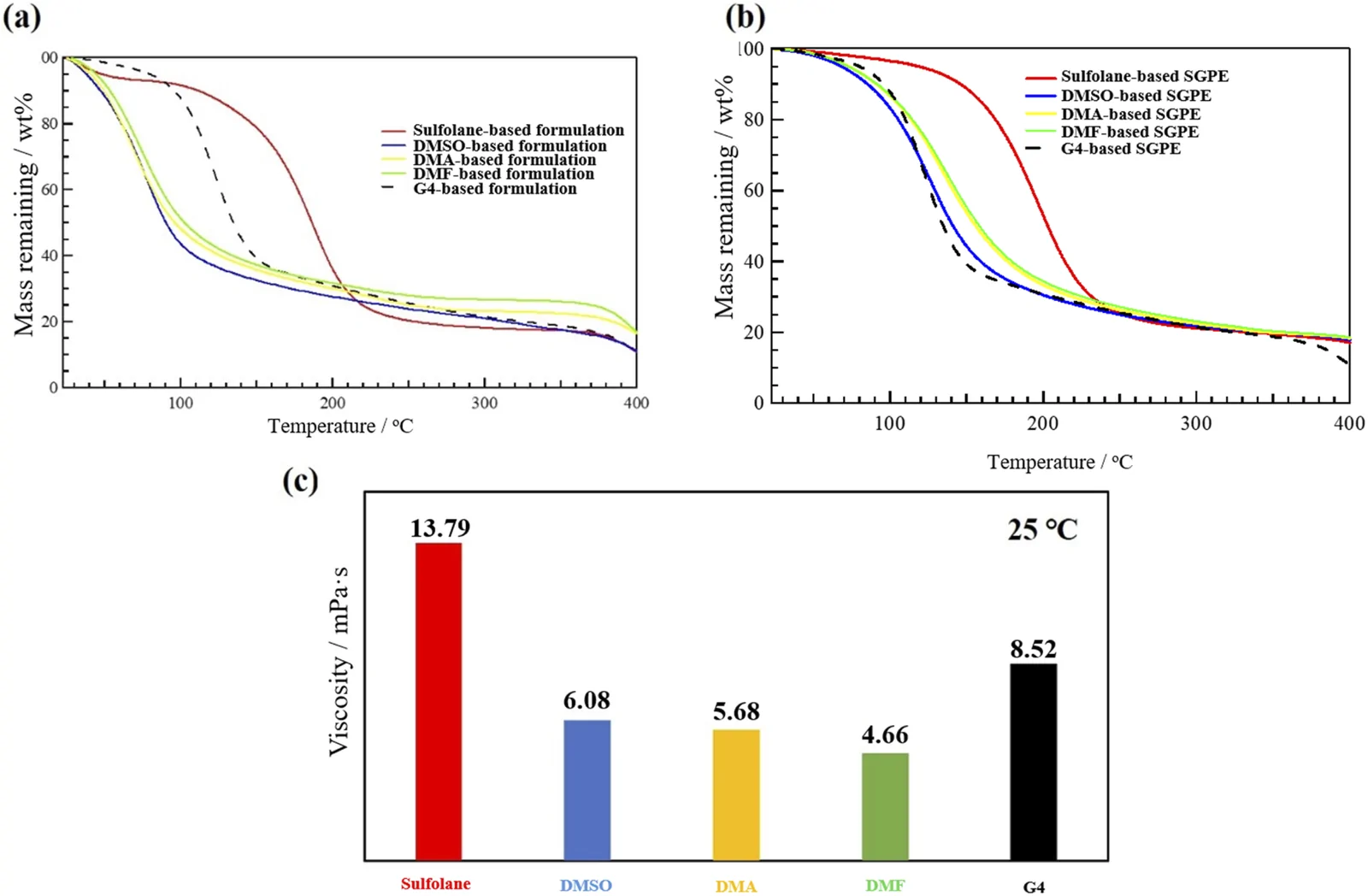
Thermogravimetric curves of (a) the liquid electrolytes and (b) the corresponding SGPEs, with the G4-based electrolyte included as a reference. (c) Dynamic viscosities of the liquid electrolyte formulations measured at 25 °C. The sulfolane-, DMSO-, DMA-, and DMF-based formulations contain 1 M LiTFSI and 10 wt% TTE.

Fig. 4(c) and Table S2 present the dynamic viscosities of the liquid electrolyte formulations containing LiTFSI and TTE, measured at 25 °C. These formulation values are distinguished from the neat-solvent properties listed in Table S1. The viscosity followed the order: sulfolane-based formulation (13.79 mPa s) > G4-based formulation (8.52 mPa s) > DMSO-based formulation (6.08 mPa s) > DMA-based formulation (5.68 mPa s) > DMF-based formulation (4.66 mPa s), with the sulfolane-based electrolyte exhibiting the highest value. Because these viscosities were measured for the complete liquid formulations containing LiTFSI and TTE, they represent formulation-level transport-related properties rather than the intrinsic viscosities of the neat solvents. Salt addition and mixing can modify viscosity through changes in intermolecular interactions and ion association. The results therefore demonstrate that solvent substitution within the common LiTFSI/TTE formulation produces substantial differences in liquid-phase viscosity, with the sulfolane-based formulation exhibiting more than twice the viscosity of the DMF-based formulation and the DMSO-based formulation showing an intermediate value.

Taken together, the TGA and viscosity measurements demonstrate distinct solvent-dependent physicochemical profiles within the common electrolyte framework. The sulfolane-based system combined comparatively high mass retention in the lower-temperature region with the highest liquid-formulation viscosity, whereas the DMSO-, DMA-, and DMF-based formulations exhibited lower viscosities but greater mass loss in the corresponding TGA region. These measurements characterize different aspects of the electrolyte formulations and do not establish a direct causal relationship between viscosity and thermal mass retention. Because all formulations contained TTE and no corresponding TTE-free controls were examined, the individual contribution of TTE to these properties cannot be separated from the solvent-dependent effects.

### 3.4 Evaluation of anodic oxidation behavior

Because Li–O_2_ batteries can reach relatively high potentials during charging, resistance of the electrolyte to anodic oxidation is an important consideration. To investigate the apparent anodic oxidation onset, linear sweep voltammetry (LSV) measurements were conducted on both liquid electrolytes and SGPEs utilizing various solvents. Fig. 5(a) and (b) display the LSV curves recorded in the potential range of 1.0–6.0 V *vs.* Li/Li^+^. For the liquid electrolytes (Fig. 5(a)), the apparent anodic oxidation onset was identified from the pronounced increase in anodic current. The onset potentials followed the order: sulfolane (5.1 V) > DMA (4.8 V) > DMSO (4.5 V) > DMF (3.3 V). The higher apparent oxidation onset of the sulfolane-based liquid formulation indicates a solvent-dependent difference in anodic behavior under the present measurement conditions. Regarding the gelled counterparts (Fig. 5(b)), the apparent anodic oxidation onsets were 5.5 V (sulfolane-based), 5.0 V (DMA-based), 4.7 V (DMSO-based), and 4.2 V (DMF-based). Under the employed LSV conditions, all SGPEs showed higher apparent anodic oxidation onsets than their corresponding liquid electrolytes. The observed shifts upon gelation indicate that incorporation of the electrolyte components into the polymer-containing system alters the anodic current response. Possible contributions include changes in molecular interactions, local solvent environment, and mass transport; however, their individual contributions cannot be distinguished from the present LSV measurements.

**Fig. 5:**
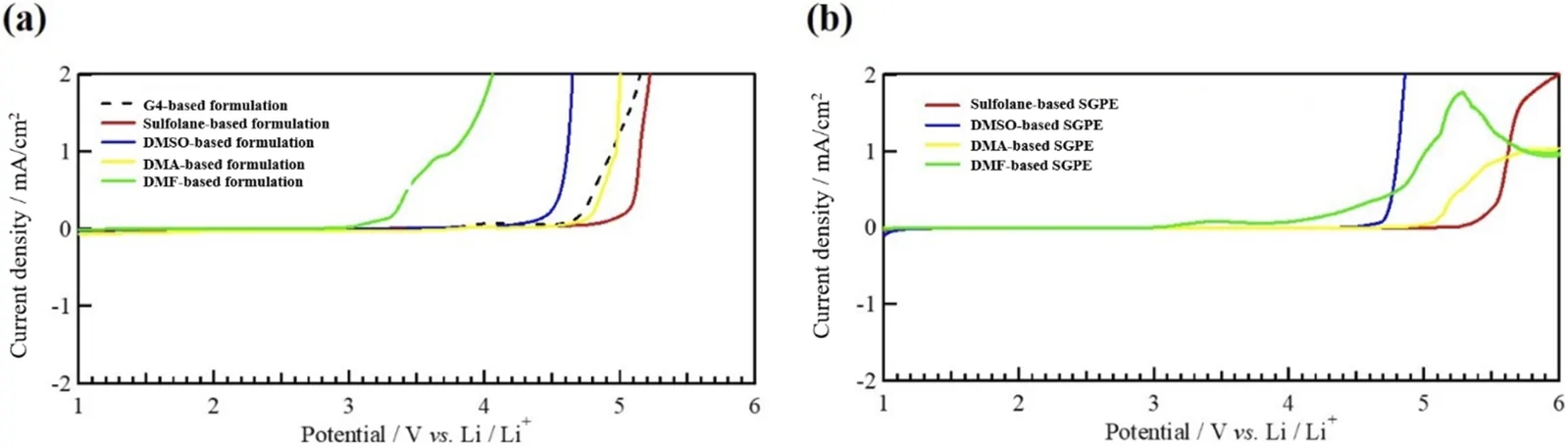
Linear sweep voltammograms of (a) liquid electrolyte formulations and (b) SGPEs prepared using different solvents. Measurements were performed over a potential range of 1.0–6.0 V *vs.* Li/Li^+^ at a scan rate of 0.1 mV s^−1^.

The sulfolane-based SGPE exhibited the highest apparent anodic oxidation onset among the tested formulations. Although the measured onset depends on the electrode configuration and measurement conditions and should not be regarded as a thermodynamic electrochemical stability limit, the result demonstrates a clear solvent-dependent difference in anodic behavior within the common SGPE framework. Together with the physicochemical differences identified in Section 3.3, this result further demonstrates that solvent substitution modifies multiple properties of the SGPE formulations. The comparatively high apparent anodic oxidation onset of the sulfolane-based SGPE is also consistent with the longer capacity-limited cycling observed in Section 3.6; however, the present measurements do not establish anodic oxidation resistance as the sole or dominant factor governing cycling performance.

### 3.5 Lithium ion transport properties in SGPEs

To evaluate Li^+^ transport characteristics within the SGPEs, the apparent Li^+^ transference number (*t*_Li^+^_) was determined using the potentiostatic polarization method (Bruce–Vincent method) with lithium symmetric cells (Li|SGPE|Li). Fig. 6 presents the chronoamperometry curves (*I*–*t* curves) and the electrochemical impedance spectroscopy (EIS) plots before and after polarization for the SGPEs prepared with each solvent (sulfolane, DMSO, DMA, and DMF). By applying the obtained steady-state current and interfacial resistance values to the Bruce–Vincent equation (eqn (2)), the *t*_Li^+^_ values were calculated. Among the formulations for which the calculation could be reliably applied, the apparent (*t*_Li^+^_) values followed the order DMA (0.66) > DMSO (0.45) > sulfolane (0.30). In contrast, for the DMF-based system, the interfacial resistance increased drastically after polarization, as shown in Fig. 6(d), preventing reliable determination of the apparent (*t*_Li^+^_) under the present measurement conditions. The pronounced increase in interfacial resistance indicates substantial interfacial instability in the DMF-containing Li|SGPE|Li cell. Parasitic reactions at lithium/electrolyte interfaces and formation of resistive interphase products are known to increase polarization resistance in gel-polymer-electrolyte systems.^32^ However, the composition of the interphase formed in the present DMF-based system was not directly analyzed. Therefore, the origin of the pronounced resistance increase cannot be assigned from the present measurements.

**Fig. 6:**
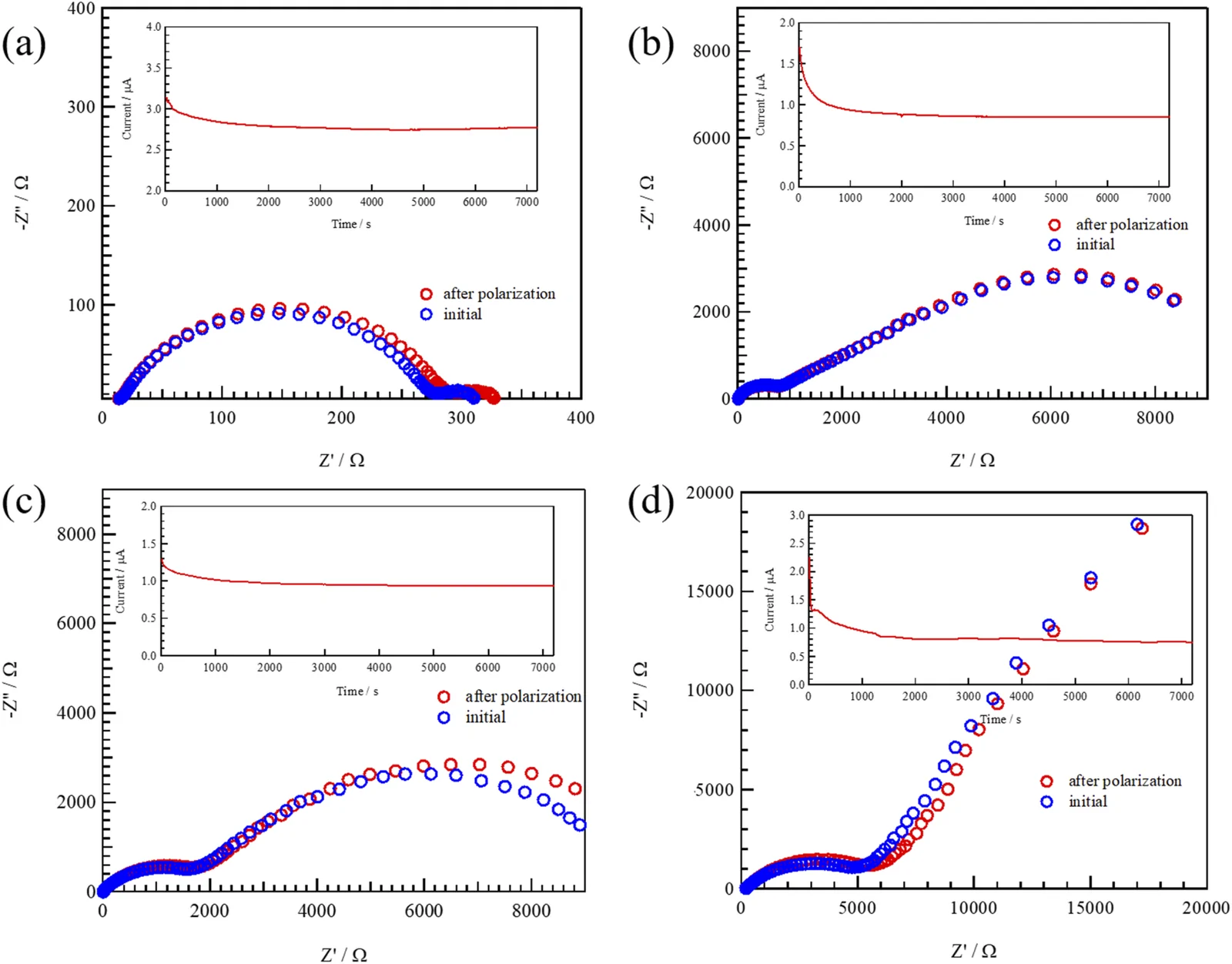
Chronoamperometry curves (*I*–*t* curves) and the Nyquist plots before and after polarization for the SGPEs prepared with 1 M LiTFSI in (a) sulfolane, (b) DMSO, (c) DMA, and (d) DMF with 10 wt% TTE SGPE using lithium symmetric cells (Li|SGPE|Li).

TTE was included as a common fluorinated component in all the investigated formulations. Its presence may affect both liquid-phase properties and reactions at the lithium interface; however, no matched TTE-free formulations or direct analyses of the lithium interphase were examined. Consequently, the present results do not distinguish the contribution of TTE to bulk transport from its possible contribution to interfacial behavior. In particular, formation of a TTE-derived LiF-rich interphase cannot be concluded from the present data.

As described in Section 2.3, the effective transport thickness of the gel-filled separator was not independently determined; therefore, absolute ionic conductivity values were not calculated. Although the separator type and precursor-solution volume were kept constant, differences in separator compression, electrolyte distribution, and electrode contact may influence the impedance response. Accordingly, the EIS results are discussed here as cell-level electrochemical responses under the present assembly conditions rather than as direct measures of the intrinsic ionic conductivity of the SGPE materials. The EIS results should therefore not be used to establish an intrinsic ionic-conductivity ranking among the SGPEs.

For the sulfolane-, DMSO-, and DMA-based formulations, the reported apparent Li^+^ transference numbers increased in the order sulfolane (0.30) < DMSO (0.45) < DMA (0.66), which broadly corresponds to decreasing liquid-formulation viscosity among these three systems (13.79, 6.08, and 5.68 mPa s, respectively; Fig. 4(c)). This association does not establish a causal relationship between viscosity and transference number. In particular, *t*_Li^+^_ represents the relative contribution of Li^+^ to ionic charge transport under the employed polarization conditions and is distinct from absolute ionic conductivity or Li^+^ diffusivity. Lower-viscosity solvents can facilitate ionic and segmental mobility in gel polymer electrolytes through plasticization of the polymer-containing phase.^33^ In addition to viscosity, differences in Li^+^-solvent coordination, Li^+^-anion association, and interactions with the polymer matrix may contribute to the observed solvent-dependent transport behavior. Their individual contributions, however, cannot be resolved from the present measurements.

Notably, the sulfolane-based SGPE exhibited the lowest apparent *t*_Li^+^_ among the three quantitatively evaluated formulations, while showing comparatively high thermal mass retention (Fig. 4) and the highest apparent anodic oxidation onset (Fig. 5). Furthermore, as discussed in Section 3.6, the sulfolane-based formulation showed the longest capacity-limited cycling among the tested systems despite its lower apparent *t*_Li^+^_. These results indicate that a higher apparent Li^+^ transference number alone did not correspond to longer capacity-limited cycling in the present SGPE system, supporting the need to consider multiple transport, stability, and interfacial properties when comparing solvent formulations.

### 3.6 Charge–discharge performance and cycle life assessment

Li–O_2_ cells incorporating SGPEs with various solvents were assembled, and galvanostatic charge–discharge tests were conducted under an oxygen atmosphere. Fig. 7(a) presents the capacity-limited cycling behavior at a current density of 0.05 mA cm^−2^ with an imposed capacity limit of 500 mAh g^−1^. The systems utilizing the sulfolane- and DMSO-based SGPEs exhibited longer cycling in the reported measurements compared with those using amide-based solvents (DMA and DMF). The DMA- and DMF-based cells showed an early decline in discharge capacity within the first few cycles. Interfacial reactions and electrolyte degradation are possible contributors to the earlier capacity loss of the DMA- and DMF-based cells. However, the present measurements do not isolate their relative importance or establish a common failure mechanism for both amide-based formulations. In particular, the pronounced interfacial resistance increase observed for the DMF-based Li|SGPE|Li symmetric cell (Fig. 6(d)) cannot by itself establish that the same interfacial process governs the capacity loss of the DMA- or DMF-based Li–O_2_ cells. Among the tested formulations, the sulfolane-based SGPE showed the longest capacity-limited cycling. The sulfolane-based cell (Fig. 7(c)) maintained discharge capacities above 400 mAh g^−1^ through 30 cycles in the reported cycling trace. The higher apparent anodic oxidation onset of the sulfolane-based SGPE is consistent with its longer cycling under the tested conditions, but this association does not establish anodic electrolyte oxidation as the sole or dominant factor controlling cycle life. The sulfolane-based cell also exhibited non-monotonic variations in discharge capacity during extended cycling, including temporary decreases followed by partial recovery. Because the origin of these variations cannot be identified from the present measurements, they are not interpreted as evidence of self-healing, interfacial recovery, or reversible structural adaptation of the SGPE. The late-stage voltage profiles (Fig. S3) show increasing discharge polarization in some cycles, and the cycle-30 profile reaches the 2.0 V cutoff before attaining 500 mAh g^−1^. Accordingly, the result is described as operation through 30 cycles with discharge capacities above 400 mAh g^−1^ in the reported trace, rather than as constant 500 mAh g^−1^ capacity retention. The reported DMSO-based cell trace, in contrast, shows a capacity decline within approximately 15 cycles (Fig. 7(a)). The lower apparent anodic oxidation onset of the DMSO-based SGPE relative to the sulfolane-based SGPE may contribute to this difference in cycling behavior at elevated charging potentials; however, the present measurements do not establish a single dominant degradation mechanism.

**Fig. 7:**
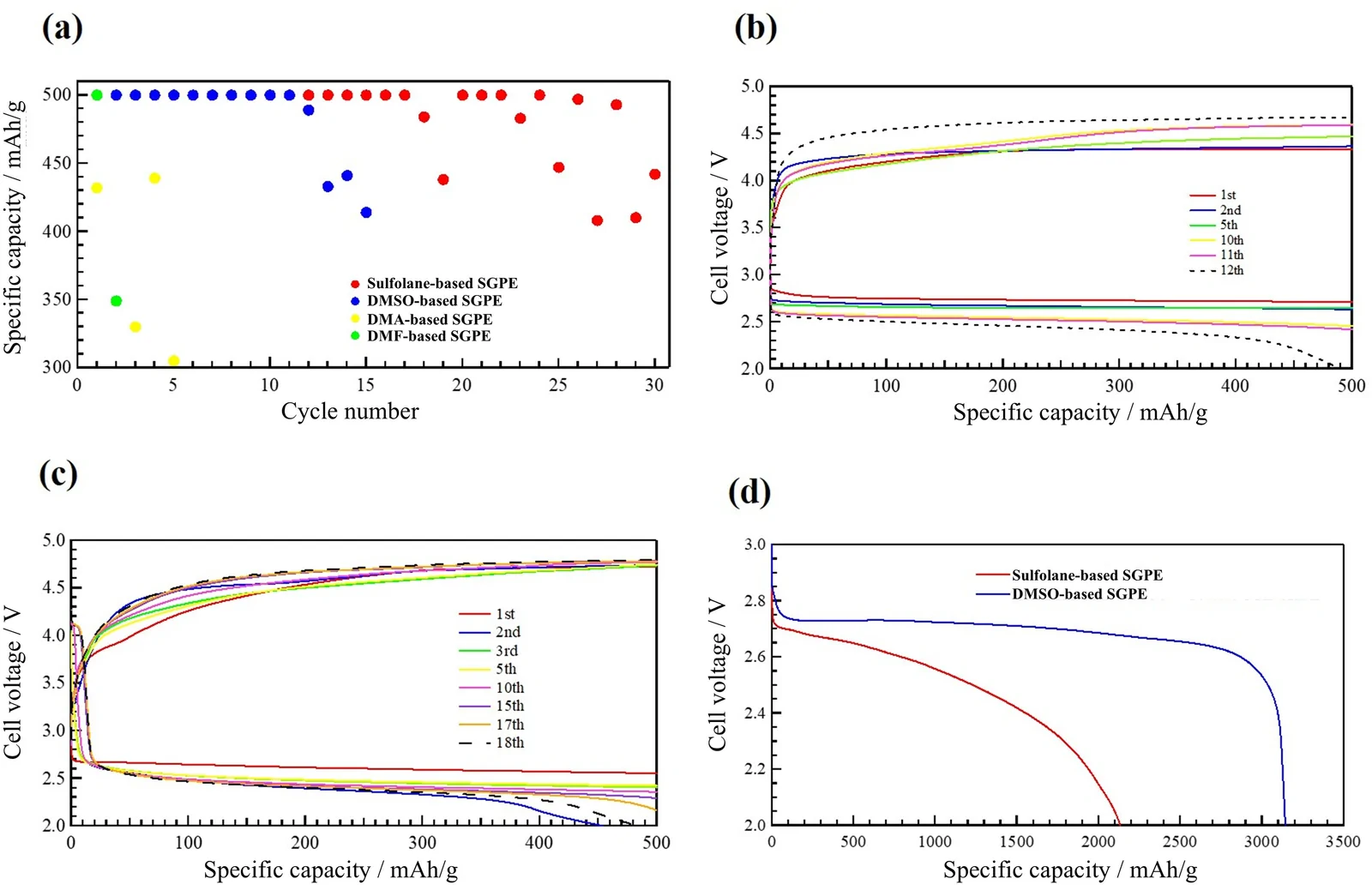
Electrochemical performance of Li–O_2_ cells employing SGPEs containing 1 M LiTFSI and 10 wt% TTE. (a) Discharge specific capacity as a function of cycle number for the sulfolane-, DMSO-, DMA-, and DMF-based SGPEs at 0.05 mA cm^−2^, with a capacity limit of 500 mAh g^−1^ and discharge and charge voltage cutoffs of 2.0 and 5.0 V, respectively. Representative charge–discharge profiles for cells employing the (b) DMSO-based SGPE and (c) sulfolane-based SGPE. Additional late-stage profiles for the cell employing the sulfolane-based SGPE are shown in Fig. S3. (d) Full-discharge profiles for cells employing the DMSO- and sulfolane-based SGPEs at 0.05 mA cm^−2^ to a lower cutoff of 2.0 V.

Next, to compare the full-discharge behavior of the sulfolane- and DMSO-based formulations, a full discharge test down to 2.0 V was conducted (Fig. 7(d)). The DMSO-based system delivered a specific capacity of 3135 mAh g^−1^, whereas the sulfolane-based system yielded 2133 mAh g^−1^. Thus, the full-discharge capacity of the DMSO-based cell was approximately 1.5 times that of the sulfolane-based cell. Furthermore, the DMSO system exhibited a higher discharge plateau voltage (*ca.* 2.7 V), consistent with lower discharge polarization under the tested conditions. The differences in polarization and discharge capacity between the DMSO- and sulfolane-based cells (Fig. 7(d)) can be discussed in relation to the measured properties of the electrolyte formulations. The DMSO-based liquid formulation has a lower measured viscosity than the sulfolane-based formulation (6.08 *versus* 13.79 mPa s at 25 °C), which is consistent with lower viscous resistance to mass transport in the liquid formulation prior to gelation. However, this liquid-formulation viscosity does not directly represent effective transport within the gel-filled porous cathode, where electrolyte distribution, pore structure, oxygen transport, and interfacial reactions can also affect discharge behavior.

Solvent donor properties are known to influence LiO_2_ solubility and the balance between surface-associated and solution-mediated Li_2_O_2_ formation in aprotic Li–O_2_ batteries.^34^ DMSO possesses strong donor characteristics, and the combination of these donor properties with the lower measured liquid-formulation viscosity is consistent with conditions that can favor intermediate solvation and transport during discharge. The higher full-discharge capacity observed for the DMSO-based cell is therefore consistent with a greater contribution from solution-mediated Li_2_O_2_ formation.^34^ However, because dissolved intermediates and the corresponding discharge-product morphology were not directly characterized for the DMSO-based cell, this reaction pathway cannot be established from the present measurements. Donor properties and viscosity should therefore be regarded as qualitative descriptors relevant to the observed solvent dependence rather than as direct measures of intermediate diffusivity or reaction kinetics. More broadly, the importance of solvent-dependent solvation and intermediate transport has also been emphasized in other conversion-type battery chemistries, including Li–S systems.^35^ Although the specific intermediates differ from those in Li–O_2_ batteries, these studies further illustrate that solvent coordination and transport properties can jointly influence reaction pathways.

The full-discharge and capacity-limited cycling results therefore reveal divergent solvent-dependent performance profiles within the common SGPE framework. At 0.05 mA cm^−2^, the DMSO-based cell delivered the higher full-discharge capacity (3135 mAh g^−1^*versus* 2133 mAh g^−1^ for sulfolane), whereas the sulfolane-based formulation showed the longest capacity-limited cycling among the tested systems. Thus, the formulation providing the highest full-discharge capacity was not the formulation showing the longest capacity-limited cycling. This divergence indicates that maximizing full-discharge capacity and sustaining repeated cycling represent distinct solvent-selection considerations in the present SGPE system. Because the galvanostatic measurements were conducted at a single current density, these results do not establish the relative rate capabilities of the formulations.

### 3.7 Morphological and structural changes of the air cathode during cycling

To examine cathode-side changes associated with charge–discharge cycling of the sulfolane-based Li–O_2_ cell, the surface morphology of the cathode (MWCNT/CP) was examined using FE-SEM. Fig. 8 displays the SEM images of (a) the pristine electrode, (b) the electrode after the fifth discharge, and (c) the electrode after the fifth charge. The corresponding *ex situ* XRD patterns are presented in Fig. S2.

**Fig. 8:**
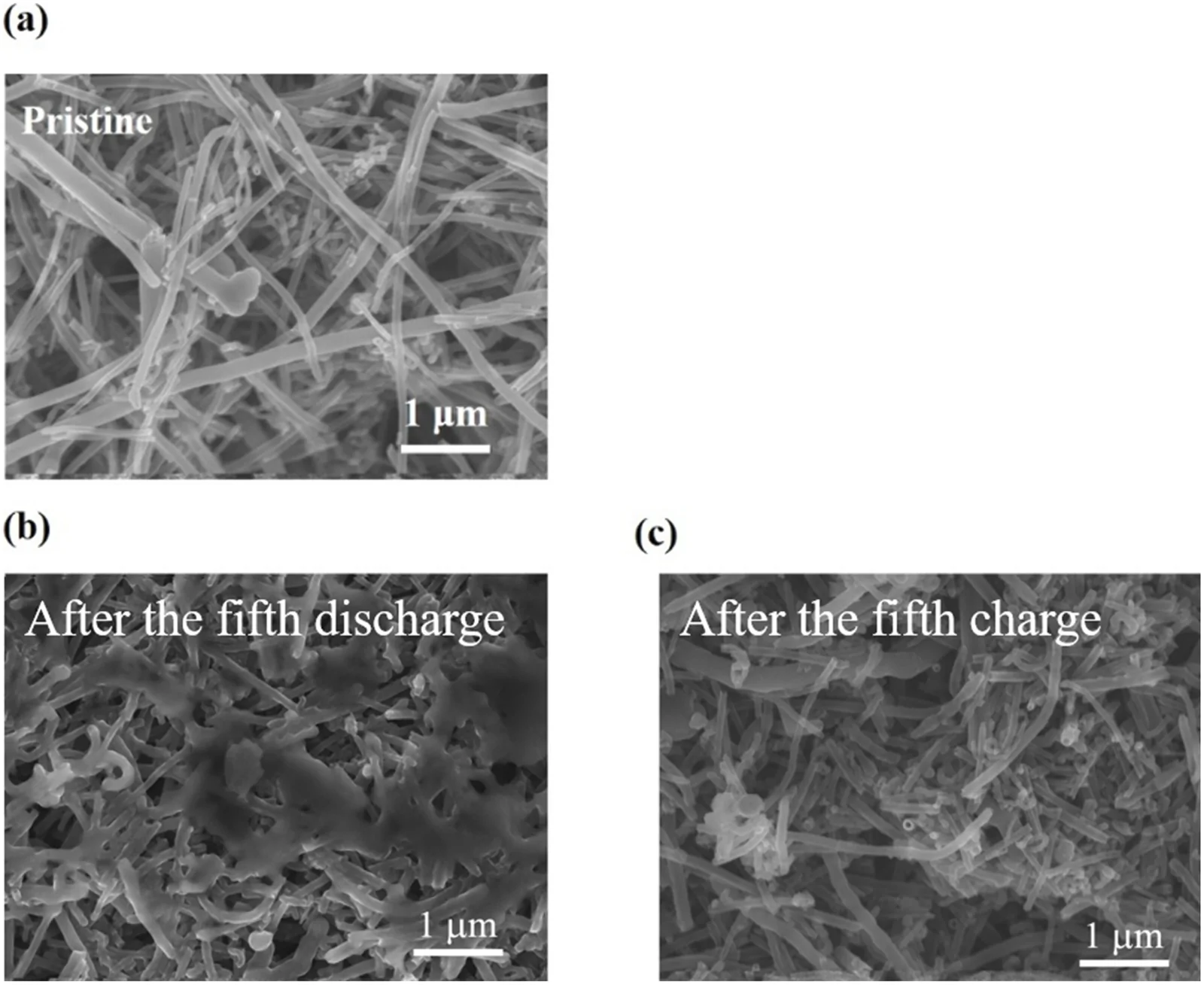
FE-SEM images of the MWCNT/carbon-paper cathode (a) before cycling, (b) after the fifth discharge, and (c) after the fifth charge in a Li–O_2_ cell employing the sulfolane-based SGPE containing 1 M LiTFSI and 10 wt% TTE.

After the fifth discharge, the MWCNT/carbon-paper cathode exhibited deposits covering portions of the fibrous network (Fig. 8(b)). The XRD pattern showed reflections assigned to crystalline Li_2_O_2_ together with weaker reflections assigned to Li_2_CO_3_ (Fig. S2). The surface deposits are consistent with partial coverage of electrochemically accessible carbon, although FE-SEM alone does not determine their chemical composition or growth pathway. Such surface coverage can reduce accessible reaction sites and impede reactant transport, thereby contributing to increased polarization and capacity limitation. After the fifth charge, the surface deposits became less prominent (Fig. 8(c)), while the reflections assigned to crystalline Li_2_O_2_ and Li_2_CO_3_ were no longer clearly resolved in the XRD pattern (Fig. S2). These changes are consistent with removal or transformation of detectable crystalline discharge products during charging. However, the absence of clearly resolved XRD reflections does not establish complete product removal or exclude residual amorphous or poorly crystalline species. Changes in the apparent MWCNT morphology were also observed after charging. Although carbon corrosion may contribute to such morphological changes, differences in the imaged regions and possible effects of electrode recovery and sample preparation prevent an unambiguous assignment of nanotube degradation from the present FE-SEM images alone. The detection of Li_2_CO_3_ after the fifth discharge should also be interpreted with caution because this electrode had undergone four preceding charge steps. Both carbon-electrode reactions and electrolyte decomposition can contribute to carbonate formation in nonaqueous Li–O_2_ cells.^36,37^ The present measurements do not distinguish these possible sources, and the observed Li_2_CO_3_ therefore cannot be assigned uniquely to either carbon-electrode degradation or electrolyte decomposition.

Taken together, the FE-SEM and XRD results demonstrate that repeated Li–O_2_ operation is accompanied by cathode-surface deposition, changes in detectable crystalline products, and morphological evolution of the carbon cathode. These cathode-side changes provide an additional factor to consider when interpreting the progressive polarization and capacity evolution observed for the sulfolane-based cell in Section 3.6. The present results do not identify a single dominant degradation mechanism; rather, they show that cathode-side product deposition and transformation accompany cycling and should be considered together with bulk electrolyte transport and lithium-interface behavior when evaluating the overall cell performance.

## 4 Conclusion

UPyMA–PETEA SGPEs containing LiTFSI and TTE were investigated using four aprotic solvents to clarify the influence of solvent selection within a common SGPE framework. Clear solvent-dependent differences were observed in liquid-formulation viscosity, thermal mass-retention behavior, apparent anodic oxidation onset, apparent Li^+^ transference number, and Li–O_2_ cell performance. At 0.05 mA cm^−2^, the DMSO-based cell delivered a higher full-discharge capacity (3135 mAh g^−1^) than the sulfolane-based cell (2133 mAh g^−1^), whereas the sulfolane-based formulation showed the longest capacity-limited cycling among the tested systems, maintaining discharge capacities above 400 mAh g^−1^ through 30 cycles in the reported trace. Thus, the formulation providing the highest full-discharge capacity was not the formulation showing the longest capacity-limited cycling. This divergence demonstrates that maximizing full-discharge capacity and sustaining repeated cycling represent distinct solvent-selection considerations in the present SGPE system.

The measured physicochemical and electrochemical properties provide context for these different performance profiles. The lower liquid-formulation viscosity and donor characteristics of DMSO are consistent with conditions favorable for transport and solution-mediated discharge, whereas the sulfolane-based SGPE exhibited comparatively high thermal mass retention and the highest apparent anodic oxidation onset among the tested formulations. These associations do not establish a single controlling solvent property or a universal solvent-selection rule, but indicate that solvent selection for Li–O_2_ SGPEs should account for multiple transport- and stability-related characteristics. Post-cycling cathode characterization further revealed surface deposition, changes in detectable crystalline products, and morphological evolution during cycling, indicating that cathode-side processes accompany the electrochemical evolution of the cell. Overall, the present results identify the balance between full-discharge capacity and sustained capacity-limited cycling as a key consideration in the solvent selection and formulation of supramolecular gel polymer electrolytes for Li–O_2_ batteries.

## Author contributions

Conceptualization and methodology, T. Is.; experimental and data analysis, T. A., T. Iw., H. T., and K. O.; writing—original draft preparation, T. A. and T. Is.; writing—review and editing, T. Is.; supervision, T. Is.; project administration and funding acquisition, T. Is. All authors have read and agreed to the published version of the manuscript.

## Conflicts of interest

There are no conflicts to declare.

## Supplementary Material

RA-016-D6RA07677G-s001

## Data Availability

The data supporting the findings of this study are available within the article and its supplementary information (SI). Supplementary information: additional experimental details, physical properties of the aprotic organic solvents, a schematic illustration of the formation of the UPyMA–PETEA polymer matrix, *ex situ* XRD patterns of the MWCNT/CP air cathode, and charge–discharge profiles of the Li–O_2_ cells. See DOI: https://doi.org/10.1039/d6ra07677g.
