# Supplementary material for: Solvent-dependent electrochemical performance of UPyMA–PETEA supramolecular gel polymer electrolytes for lithium–oxygen batteries
Source: RSC Adv. 2026 Oct 12;16(47):62504–16. doi: 10.1039/d6ra07677g (PMC13657200; doi:10.1039/d6ra07677g)
Supplement: Supplementary file 1 [file RA-016-D6RA07677G-s001.pdf]

Supplementary Information (SI) for RSC Advances.
This journal is © The Royal Society of Chemistry

Electronic Supplementary Information

Solvent-Dependent Electrochemical Performance of UPyMA–PETEA Supramolecular Gel Polymer Electrolytes for Lithium–Oxygen Batteries

Toshiki Aizawa[1], Toshiki Iwato[1], Hayato Takada[1], Kentaro Ogihara[1], Takahiro Ishizaki[2,*]

[1]Materials Science and Engineering, Graduate School of Engineering and Science, Shibaura Institute of Technology, Toyosu, Koto-Ku, Tokyo, 135-8548, Japan

[2]College of Engineering, Shibaura Institute of Technology, Toyosu, Koto-Ku, Tokyo, 135-8548, Japan

*Corresponding author: ishizaki@shibaura-it.ac.jp

S1 Experimental method

S1.1 Physicochemical Characterization

The viscosity of the electrolytes was measured at room temperature using a rotational viscometer (Visco QC100, Anton Paar). The measurement samples were prepared by dissolving 1M LiTFSI in each solvent (sulfolane, DMSO, DMF, and DMA) with the addition of 10 wt% TTE. To evaluate thermal stability, weight changes of the liquid electrolytes and SGPEs were monitored in the temperature range of 30 – 400 °C using a simultaneous thermogravimetry/differential thermal analysis instrument (TG-DTA, DTG-60H, Shimadzu). For comparison, the viscosity and thermal properties of an electrolyte based on tetraethylene glycol dimethyl ether (TEGDME, G4)—a common solvent for lithium-air batteries—were also evaluated as a benchmark.

S1.2 Preparation of Air Cathode

Polyvinylidene fluoride (PVDF) was dissolved in *N*-methyl-2-pyrrolidone (NMP) to prepare a 1 wt% PVDF solution as a binder dispersion. Commercial multi-walled carbon nanotubes (MWCNTs) were added to this solution at a MWCNT : PVDF weight ratio of 9:1. The mixture was stirred for 24 h on a hot plate stirrer, followed by ultrasonic dispersion for 3 h to obtain a homogeneous slurry. The resulting slurry was coated onto carbon paper (TGP-H-060, Toray Co.; f 15 mm) to achieve an MWCNT mass loading of 0.5 ± 0.2 mg/cm^2. The coated electrodes were dried at 120 °C under vacuum for over 24 h to remove the solvent, yielding the oxygen cathodes.

S1.3 Characterization of Discharge Products

To investigate the crystalline structure and morphology of the discharge products, the cells were disassembled in an argon-filled glovebox after the discharge test. The recovered cathodes were washed with dimethoxyethane (DME), which had been dehydrated using molecular sieves, to remove residual electrolyte components, and subsequently dried under vacuum. For X-ray diffraction (XRD) measurements, the dried cathodes were mounted onto a sealed atmosphere-protected sample holder (Rigaku) inside the argon-filled glovebox to prevent any exposure to ambient air and moisture during transfer and analysis. For surface morphology characterization, the cathodes were placed in a sealed transfer container inside the glovebox and transported to the electron microscope. Immediately before the measurement, the samples were retrieved from the container, quickly mounted onto the stage, and transferred into the sample chamber of a field-emission scanning electron microscopy (FE-SEM, JSM-7610F, JEOL) for observation.

Table S1 Literature-reported physical properties of the neat solvents used in this study.

**Solvent**	**Viscosity / mPa·s**	**Temperature for viscosity / °C**	**Reference for viscosity**
Sulfolane	10.3	30	S1
DMSO	2.0	25	S1
DMA (*N,N*-dimethylacetamide)	0.838	30	S2
DMF (*N,N*-dimethylformamide)	0.802	25	S3

Viscosity values refer to the neat solvents at the temperatures indicated and were obtained from the cited sources. These values do not represent the viscosities of the formulated electrolytes containing LiTFSI and TTE. Because the listed temperatures differ, the values should not be treated as an isothermal comparison.

Table S2 Measured dynamic viscosities of the liquid electrolyte formulations containing LiTFSI and TTE.

**Main solvent**	**LiTFSI concentration**	**TTE content**	**Measurement temperature / °C**	**Dynamic viscosity / mPa s**
sulfolane	1 M	10 wt%	25	13.79
DMSO	1 M	10 wt%	25	6.08
DMA	1 M	10 wt%	25	5.68
DMF	1 M	10 wt%	25	4.66

The viscosities were measured for the liquid electrolyte formulations described in Section S1.1, not for the neat solvents or the polymerized SGPEs. These values correspond to the measurements presented in Fig. 4c.

Fig. S1 Schematic illustration for the formation mechanism of the polymer matrix (UPyMA-PETEA).

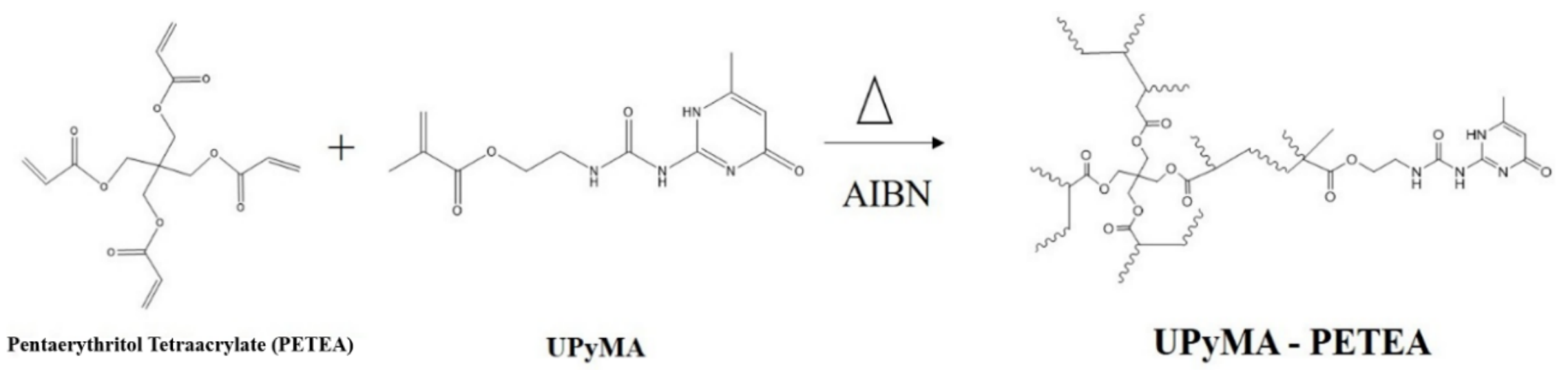

Fig. S2 *Ex situ* XRD patterns of the MWCNT/CP air cathode in the pristine state, after the 5th discharge, and after the 5th charge using the sulfolane-based SGPE (1 M LiTFSI in sulfolane + 10 wt% TTE SGPE). Stars (□) and squares (□) denote the diffraction peaks corresponding to Li_2O_2 and Li_2CO_3, respectively. Filled circles (●) represent the graphitic carbon substrate peaks (MWCNT/CP).

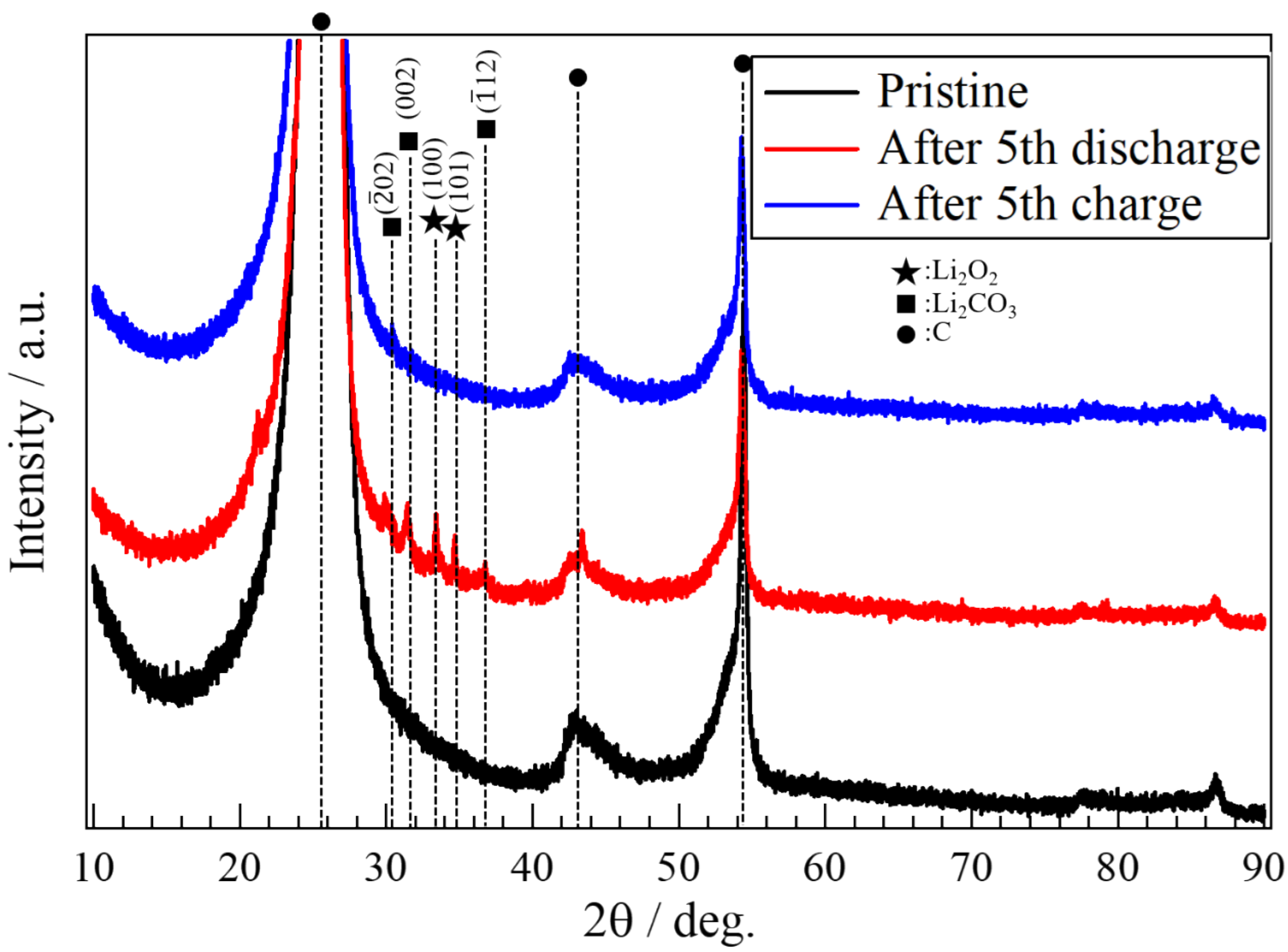

Fig. S3 Representative late-stage charge–discharge profiles of a Li–O₂ cell employing the sulfolane-based SGPE containing 1 M LiTFSI and 10 wt% TTE at a current density of 0.05 mA/cm^2 with a capacity limit of 500 mAh/g and discharge and charge voltage cutoffs of 2.0 and 5.0 V, respectively. The 20th, 24th, 28th, and 30th cycles are shown to illustrate the evolution of the cycling behavior.

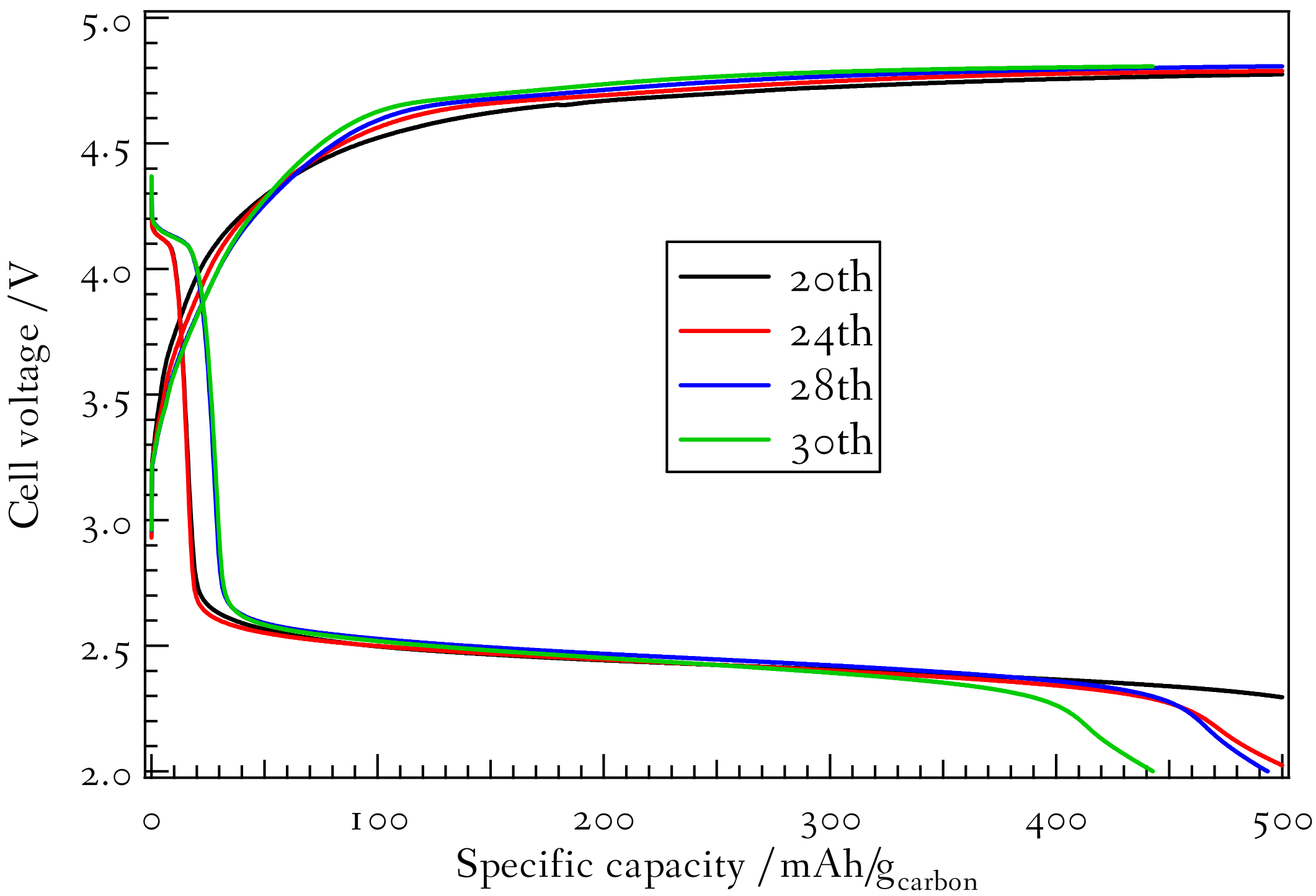

References

S1. Gaylord Chemical Company, LLC, *Dimethyl Sulfoxide Physical Properties*, Bulletin 101, Fig. 23, online PDF, (accessed 23 September 2026).

S2. Nanjing Shundajie Chemical New Materials Co., Ltd., *Basic chemical series – dimethylacetamide*, 2024, online, (accessed 23 September 2026).

S3. Eastman Chemical Company, *Dimethylformamide (DMF)*, Technical Data Sheet, online, (accessed 23 September 2026).
